# Supplementary material for: Serum Albumin: A Multifaced Enzyme
Source: Int J Mol Sci. 2021 Sep 18;22(18):10086. doi: 10.3390/ijms221810086 (PMC8466385; doi:10.3390/ijms221810086)
Supplement: Supplementary file 1 [file ijms-22-10086-s001.zip › ijms-1340117-supplementary.pdf]

Supplementary Materials

Serum albumin: a multifaced enzyme

Giovanna De Simone [a,*], Alessandra di Masi [a], Paolo Ascenzi [b,c]

[a] *Dipartimento di Scienze, Università Roma Tre, Viale Marconi 446, 00146 Roma, Italy*

[b] *Laboratorio Interdipartimentale di Microscopia Elettronica, Università Roma Tre,*

Via della Vasca Navale 79, 00146 Roma, Italy

[c] *Accademia Nazionale dei Lincei, Via della Lungara 10, 00165 Roma, Italy*

Running title: Serum albumin: a multifaced enzyme

* Corresponding Author: Dr. Giovanna De Simone, PhD, Dipartimento di Scienze, Università Roma Tre, Viale Marconi 446, 00146 Roma, Italy

e-mail: giovanna.desimone@uniroma3.it (Giovanna De Simone)

Table S1. Three dimensional structures of the HSA:ligand complexes.

PDB code	Resolution (Å)	Ligands	Ligand binding sites	References
		Crystal structures		
1AO6	2.50	Ligand free	n.a.	[1]
1BJ5	2.50	Myristic acid	FA1, FA2, FA3, FA4, FA5	[2]
1BKE	3.15	Myristic acid<br>Tri-iodobenzoic acid	FA1, FA2, FA3, FA4, FA5<br>FA1[a], FA7	[2]
1BM0	2.50	Ligand free	n.a.	[1]
1E78	2.60	Ligand free	n.a.	[3]
1E7A	2.20	Propofol	FA3–FA4, FA5	[3]
1E7B	2.38	Halothane	FA3, FA6	[3]
1E7C	2.40	Myristic acid<br>Halothane	FA1, FA2, FA3, FA4, FA5<br>FA2, FA6, FA7, IA/IB	[3]
1E7E	2.50	Capric acid	FA1, FA2, FA3, FA4, FA5, FA6[b], FA7, FA8, FA9	[4]
1E7F	2.43	Lauric acid	FA1, FA2[b], FA3, FA4, FA5, FA6, FA7	[4]
1E7G	2.50	Myristic acid	FA1, FA2, FA3, FA4, FA5, FA6, FA7	[4]
1E7H	2.43	Palmitic acid	FA1, FA2, FA3, FA4, FA5, FA6, FA7	[4]
1E7I	2.70	Stearic acid	FA1, FA2, FA3, FA4, FA5, FA6, FA7	[4]
1GNI	2.40	Oleic acid	FA1, FA2, FA3, FA4, FA5, FA6, FA7	[5]
1GNJ	2.60	Arachidonic acid	FA1, FA2, FA3, FA4, FA5, FA6, FA7	[5]
1H9Z	2.50	Myristic acid<br>R-(+) warfarin enantiomer	FA1, FA2, FA3, FA4, FA5, FA6<br>FA7	[5]
1HA2	2.50	Myristic acid<br>*S*-(–) warfarin enantiomer	FA1, FA2, FA3, FA4, FA5, FA6<br>FA7	[5]
1HK1	2.65	Thyroxine	Tr-1[c], Tr-2[d], Tr-3[e], Tr-4[f]	[6]
1HK2[g]	2.80	Thyroxine	Tr-1[c], Tr-2[d], Tr-3[e], Tr-4[f]	[6]
1HK3[h]	2.80	Thyroxine	Tr-1[c], Tr-2[d], Tr-3[e], Tr-4[f]	[6]
1HK4	2.40	Myristic acid<br>Thyroxine	FA1, FA2, FA3, FA4, FA5, FA6, FA7<br>Tr-5[i]	[6]
1HK5	2.70	Myristic acid<br>Thyroxine	FA1, FA2, FA3, FA4, FA5, FA6, FA7<br>Tr-5[i]	[6]
1N5U	1.90	Myristic acid<br>Heme	FA2, FA3, FA4, FA5, FA6<br>FA1	[7]
1O9X	3.20	Myristic acid<br>Heme	FA2, FA3, FA4, FA5, FA6, FA7<br>FA1	[8]
1TF0	2.70	Citric acid<br>Decanoic acid<br>GA module of *P. magnus* PAB	FA7[a]<br>FA6[b], FA7<br>IIA/IIB	[9]
1UOR	2.80	Ligand free	n.a.	[10]
2BX8	2.70	Azapropazone	FA1[a], FA7	[11]
2BXA	2.35	3-carboxy-4-methyl-5-propyl- 2-furanpropanoic acid	FA3–FA4, FA7	[11]
2BXB	3.20	Oxyphenbutazone	FA7	[11]
2BXC	3.10	Phenylbutazone	FA7	[11]
2BXD	3.05	Warfarin	FA7	[11]
2BXE	2.95	Diflunisal	FA3–FA4, FA6, FA7	[11]
2BXF	2.95	Diazepam	FA3–FA4	[11]
2BXG	2.70	Ibuprofen	FA3–FA4, FA6	[11]
2BXH	2.25	Indoxyl sulfate	FA3–FA4, FA7[a]	[11]
2BXI	2.50	Myristic acid<br>Azapropazone	FA2, FA3, FA4, FA5, FA6<br>FA1, FA7	[11]
2BXK	2.40	Myristic acid<br>Azapropazone<br>Indomethacin	FA1, FA2, FA3, FA4, FA5, FA6<br>FA7<br>FA7[a]	[11]
2BXL	2.60	Myristic acid<br>3,5-diiodosalicylic acid	FA1, FA2, FA3, FA4, FA5, FA6, FA7<br>FA7	[11]
2BXM	2.50	Myristic acid<br>Indomethacin	FA1, FA2, FA3, FA4, FA5, FA6, FA7<br>FA1, FA7[a]	[11]
2BXN	2.65	Myristic acid<br>Iodipamide	FA1, FA2, FA3, FA4, FA5, FA6<br>FA7[b], FA9	[11]
2BXO	2.60	Myristic acid<br>Oxyphenbutazone	FA1, FA2, FA3, FA4, FA5, FA6<br>FA5[a], FA7	[11]
2BXP	2.30	Myristic acid	FA1, FA2, FA3–FA4, FA5, FA6, FA8	[11]

		Phenylbutazone	FA7	
2BXQ	2.60	Myristic acid<br>Phenylbutazone<br>Indomethacin	FA2, FA3, FA4, FA5, FA6<br>FA7<br>FA1, FA8	[11]
2I2Z	2.70	Myristic acid<br>Aspirin	FA1, FA2, FA3, FA4, FA5<br>FA7	[12]
2I30	2.90	Myristic acid<br>Salicylic acid	FA1[l], FA2, FA3, FA4, FA5<br>FA1[l], FA7	[12]
2VDB	2.52	Capric acid<br>*S*-Naproxen<br>GA module of *P. magnus* PAB	FA1, FA2, FA3, FA4, FA6, FA7<br>FA1<br>IIA, IIB	[13]
2VUE	2.42	4Z,15E-bilirubin-IX-a	FA1	[14]
2VUF	3.05	Fusidic acid	FA1, Tr-4[f]	[14]
2XSI	2.70	Myristic acid<br>Dansyl-L-glutamate	FA1, FA2, FA3, FA4, FA5, FA6<br>FA1, FA7	[15]
2XVQ	2.90	Dansyl-L-sarcosine	FA3–FA4	[15]
2XVU	2.60	Dansyl-L-asparagine	FA3–FA4, FA7	[15]
2XVV	2.40	Myristic acid<br>Dansyl-L-asparagine	FA1, FA2, FA3, FA4, FA5, FA6<br>FA1, FA7	[15]
2XVW	2.65	Myristic acid<br>Dansyl-L-arginine	FA1, FA2, FA3, FA4, FA5, FA6<br>FA1, FA7	[15]
2XW0	2.40	Dansyl-L-phenylalanine	FA3–FA4, FA7	[15]
2XW1	2.50	Dansyl-L-norvaline	FA3–FA4	[15]
2YDF	2.75	Iophenoxic acid	FA1[a], FA3–FA4, FA7, IIIA-IIIB	[16]
3A73	2.19	Myristic acid<br>D12-prostaglandin J2	FA2, FA3, FA4, FA5, FA6, FA7<br>FA1[b]	[17]
3B9L	2.60	Myristic acid<br>AZT	FA1, FA2, FA3, FA4, FA5, FA6, FA7<br>FA1, FA7	[18]
3B9M	2.70	Myristic acid<br>AZT<br>Salycilic acid	FA1, FA2, FA3, FA4, FA5, FA6<br>FA7<br>FA1[l], FA7	[18]
3CX9	2.80	Myristic acid<br>Lisophosphatidylethanilamine	FA2, FA3, FA4, FA5, FA6<br>FA7	[19]
3JQZ	3.30	Lidocaine	FA9	[20]
3JRY	2.30	Sulfate	IIIA	[20]
3LU6	2.70	Compound 1[m]	FA6, FA7	[21]
3LU7	2.80	Compound 2[n]	FA7	[21]
3LU8	2.60	Compound 3[o]	FA1	[21]
3SQJ	2.05	Myristic acid	FA1, FA2, FA3, FA4, FA5, FA6, FA7	[22]
3UIV	2.20	Amantadine	FA7	*unpublished*
4E99	2.30	Perfluorooctane sulfonate	IIA, IIB, IIIA	[23]
4L8U	2.01	9-Aminocamptothecin	FA1	[24]
4LA0	2.40	Bicalutamide	FA1	[24]
4L9K	2.40	Camptothecin	FA1	[24]
4S1Y	3.16	Cisplatin	IA, IIB	[25]
4LB9	2.70	Etoposide	FA7	[24]
4LB2	2.80	Idarubicin	FA1	[24]
4L9Q	2.70	Teniposide	FA1	[24]
4Z69	2.19	Diclofenac	FA1, FA7	[26]
5GIX	2.80	Palmitic acid-Fe(Hn$_3$piT)Cl$_2$	IIA	[27]
5X52	3.00	Octanoate<br>N-acetyl-L-methionine	FA3<br>FA7	[28]
5YOQ	2.65	4-Phenylbutyrate	FA3-FA4	[29]
6A7P	2.28	Aripiprazole	FA3-FA4	[30]
6XV0	3.00	Lauric acid functionalized hexamolybdoaluminate	IIIA, IIIB, connection loop of subdomains IA and IB	[31]
7AAE	2.27	Myristic acid<br>Perfluorooctanoic	FA1, FA2, FA3, FA4, FA5, FA6, FA7<br>IIIA	[32]
7D6J	3.29	Benzbromarone	IB, IIA, and IIIA	[33]
7JWN	2.60 Å	Ketoprofen	FA3-FA4, FA7	*unpublished*
Solution structures				
1YSX	n.a.	Thioethylamino-2,4-dimethylphenyl	FA3–FA4	[34]
2ESG	n.a.	Immunoglobulin IgA1	Cys34	[35]

[a] Partial occupancy.

[b] two ligand molecules in the site.

[c] Tr-1 largely corresponds to FA7. The partial occupancy of this site does not allow to say that it matches with fatty acid binding site.

[d] Tr-2 largely corresponds to FA3–FA4. The partial occupancy of these sites do not allow to say that they matches with fatty acid binding sites.

[e] Tr-3 largely corresponds to FA5. The partial occupancy of this site does not allow to say that this site matches with fatty acid binding site.

[f] Tr-4 is in the proximity of FA5.

[g] Arg218His.

[h] Arg218Phe.

[i] Tr-5 corresponds to FA9.

[l] myristic acid and salicylic acid occupy two contiguous regions forming the heme site.

[m] [(1R,2R)-2-{[(5-fluoro-1H-indol-2-yl)carbonyl]amino}-2,3-dihydro-1H-inden-1-yl]acetic acid.

[n] 4-[(1R,2R)-2-{[(5-fluoro-1H-indol-2-yl)carbonyl]amino}-2,3-dihydro-1H-inden-1-yl]butanoic acid.

[o] N-[5-(5-{[(2,4-dimethyl-1,3-thiazol-5-yl)sulfonyl]amino}-6-fluoropyridin-3-yl)-4-methyl-1,3-thiazol-2-yl]acetamide.

References

1. Sugio, S.; Kashima, A.; Mochizuki, S.; Noda, M.; Kobayashi, K. Crystal structure of human serum albumin at 2.5 A resolution. *Protein engineering* **1999**, *12*, 439-446, doi:10.1093/protein/12.6.439.
2. Curry, S.; Mandelkow, H.; Brick, P.; Franks, N. Crystal structure of human serum albumin complexed with fatty acid reveals an asymmetric distribution of binding sites. *Nature structural biology* **1998**, *5*, 827-835, doi:10.1038/1869.
3. Bhattacharya, A.A.; Curry, S.; Franks, N.P. Binding of the general anesthetics propofol and halothane to human serum albumin. High resolution crystal structures. *The Journal of biological chemistry* **2000**, *275*, 38731-38738, doi:10.1074/jbc.M005460200.
4. Bhattacharya, A.A.; Grune, T.; Curry, S. Crystallographic analysis reveals common modes of binding of medium and long-chain fatty acids to human serum albumin. *Journal of molecular biology* **2000**, *303*, 721-732, doi:10.1006/jmbi.2000.4158.
5. Petitpas, I.; Grune, T.; Bhattacharya, A.A.; Curry, S. Crystal structures of human serum albumin complexed with monounsaturated and polyunsaturated fatty acids. *Journal of molecular biology* **2001**, *314*, 955-960, doi:10.1006/jmbi.2000.5208.
6. Petitpas, I.; Petersen, C.E.; Ha, C.E.; Bhattacharya, A.A.; Zunszain, P.A.; Ghuman, J.; Bhagavan, N.V.; Curry, S. Structural basis of albumin-thyroxine interactions and familial dysalbuminemic hyperthyroxinemia. *Proceedings of the National Academy of Sciences of the United States of America* **2003**, *100*, 6440-6445, doi:10.1073/pnas.1137188100.
7. Wardell, M.; Wang, Z.; Ho, J.X.; Robert, J.; Ruker, F.; Ruble, J.; Carter, D.C. The atomic structure of human methemalbumin at 1.9 A. *Biochemical and biophysical research communications* **2002**, *291*, 813-819, doi:10.1006/bbrc.2002.6540.
8. Zunszain, P.A.; Ghuman, J.; Komatsu, T.; Tsuchida, E.; Curry, S. Crystal structural analysis of human serum albumin complexed with hemin and fatty acid. *BMC structural biology* **2003**, *3*, 6, doi:10.1186/1472-6807-3-6.
9. Lejon, S.; Frick, I.M.; Bjorck, L.; Wikstrom, M.; Svensson, S. Crystal structure and biological implications of a bacterial albumin binding module in complex with human serum albumin. *The Journal of biological chemistry* **2004**, *279*, 42924-42928, doi:10.1074/jbc.M406957200.
10. He, X.M.; Carter, D.C. Atomic structure and chemistry of human serum albumin. *Nature* **1992**, *358*, 209-215, doi:10.1038/358209a0.
11. Ghuman, J.; Zunszain, P.A.; Petitpas, I.; Bhattacharya, A.A.; Otagiri, M.; Curry, S. Structural basis of the drug-binding specificity of human serum albumin. *Journal of molecular biology* **2005**, *353*, 38-52, doi:10.1016/j.jmb.2005.07.075.
12. Yang, F.; Bian, C.; Zhu, L.; Zhao, G.; Huang, Z.; Huang, M. Effect of human serum albumin on drug metabolism: structural evidence of esterase activity of human serum albumin. *Journal of structural biology* **2007**, *157*, 348-355, doi:10.1016/j.jsb.2006.08.015.
13. Lejon, S.; Cramer, J.F.; Nordberg, P. Structural basis for the binding of naproxen to human serum albumin in the presence of fatty acids and the GA module. *Acta crystallographica. Section F, Structural biology and crystallization communications* **2008**, *64*, 64-69, doi:10.1107/S174430910706770X.
14. Zunszain, P.A.; Ghuman, J.; McDonagh, A.F.; Curry, S. Crystallographic analysis of human serum albumin complexed with 4Z,15E-bilirubin-IXalpha. *Journal of molecular biology* **2008**, *381*, 394-406, doi:10.1016/j.jmb.2008.06.016.
15. Ryan, A.J.; Ghuman, J.; Zunszain, P.A.; Chung, C.W.; Curry, S. Structural basis of binding of fluorescent, site-specific dansylated amino acids to human serum albumin. *Journal of structural biology* **2011**, *174*, 84-91, doi:10.1016/j.jsb.2010.10.004.
16. Ryan, A.J.; Chung, C.W.; Curry, S. Crystallographic analysis reveals the structural basis of the high-affinity binding of iophenoxic acid to human serum albumin. *BMC structural biology* **2011**, *11*, 18, doi:10.1186/1472-6807-11-18.

17. Yamaguchi, S.; Aldini, G.; Ito, S.; Morishita, N.; Shibata, T.; Vistoli, G.; Carini, M.; Uchida, K. Delta12-prostaglandin J2 as a product and ligand of human serum albumin: formation of an unusual covalent adduct at His146. *Journal of the American Chemical Society* **2010**, *132*, 824-832, doi:10.1021/ja908878n.
18. Zhu, L.; Yang, F.; Chen, L.; Meehan, E.J.; Huang, M. A new drug binding subsite on human serum albumin and drug-drug interaction studied by X-ray crystallography. *Journal of structural biology* **2008**, *162*, 40-49, doi:10.1016/j.jsb.2007.12.004.
19. Guo, S.; Shi, X.; Yang, F.; Chen, L.; Meehan, E.J.; Bian, C.; Huang, M. Structural basis of transport of lysophospholipids by human serum albumin. *The Biochemical journal* **2009**, *423*, 23-30, doi:10.1042/BJ20090913.
20. Hein, K.L.; Kragh-Hansen, U.; Morth, J.P.; Jeppesen, M.D.; Otzen, D.; Moller, J.V.; Nissen, P. Crystallographic analysis reveals a unique lidocaine binding site on human serum albumin. *Journal of structural biology* **2010**, *171*, 353-360, doi:10.1016/j.jsb.2010.03.014.
21. Buttar, D.; Colclough, N.; Gerhardt, S.; MacFaul, P.A.; Phillips, S.D.; Plowright, A.; Whittamore, P.; Tam, K.; Maskos, K.; Steinbacher, S.; et al. A combined spectroscopic and crystallographic approach to probing drug-human serum albumin interactions. *Bioorganic & medicinal chemistry* **2010**, *18*, 7486-7496, doi:10.1016/j.bmc.2010.08.052.
22. He, Y.; Ning, T.; Xie, T.; Qiu, Q.; Zhang, L.; Sun, Y.; Jiang, D.; Fu, K.; Yin, F.; Zhang, W.; et al. Large-scale production of functional human serum albumin from transgenic rice seeds. *Proceedings of the National Academy of Sciences of the United States of America* **2011**, *108*, 19078-19083, doi:10.1073/pnas.1109736108.
23. Luo, Z.; Shi, X.; Hu, Q.; Zhao, B.; Huang, M. Structural evidence of perfluorooctane sulfonate transport by human serum albumin. *Chemical research in toxicology* **2012**, *25*, 990-992, doi:10.1021/tx300112p.
24. Wang, Z.M.; Ho, J.X.; Ruble, J.R.; Rose, J.; Ruker, F.; Ellenburg, M.; Murphy, R.; Click, J.; Soistman, E.; Wilkerson, L.; et al. Structural studies of several clinically important oncology drugs in complex with human serum albumin. *Biochimica et biophysica acta* **2013**, *1830*, 5356-5374, doi:10.1016/j.bbagen.2013.06.032.
25. Ferraro, G.; Massai, L.; Messori, L.; Merlino, A. Cisplatin binding to human serum albumin: a structural study. *Chemical communications* **2015**, *51*, 9436-9439, doi:10.1039/c5cc01751c.
26. Zhang, Y.; Lee, P.; Liang, S.; Zhou, Z.; Wu, X.; Yang, F.; Liang, H. Structural basis of non-steroidal anti-inflammatory drug diclofenac binding to human serum albumin. *Chemical biology & drug design* **2015**, *86*, 1178-1184, doi:10.1111/cbdd.12583.
27. Qi, J.; Gou, Y.; Zhang, Y.; Yang, K.; Chen, S.; Liu, L.; Wu, X.; Wang, T.; Zhang, W.; Yang, F. Developing Anticancer Ferric Prodrugs Based on the N-Donor Residues of Human Serum Albumin Carrier IIA Subdomain. *Journal of medicinal chemistry* **2016**, *59*, 7497-7511, doi:10.1021/acs.jmedchem.6b00509.
28. Kawai, A.; Chuang, V.T.G.; Kouno, Y.; Yamasaki, K.; Miyamoto, S.; Anraku, M.; Otagiri, M. Crystallographic analysis of the ternary complex of octanoate and N-acetyl-l-methionine with human serum albumin reveals the mode of their stabilizing interactions. *Biochimica et biophysica acta. Proteins and proteomics* **2017**, *1865*, 979-984, doi:10.1016/j.bbapap.2017.04.004.
29. Kawai, A.; Yamasaki, K.; Enokida, T.; Miyamoto, S.; Otagiri, M. Crystal structure analysis of human serum albumin complexed with sodium 4-phenylbutyrate. *Biochemistry and biophysics reports* **2018**, *13*, 78-82, doi:10.1016/j.bbrep.2018.01.006.
30. Sakurama, K.; Kawai, A.; Tuan Giam Chuang, V.; Kanamori, Y.; Osa, M.; Taguchi, K.; Seo, H.; Maruyama, T.; Imoto, S.; Yamasaki, K.; et al. Analysis of the Binding of Aripiprazole to Human Serum Albumin: The Importance of a Chloro-Group in the Chemical Structure. *ACS omega* **2018**, *3*, 13790-13797, doi:10.1021/acsomega.8b02057.

31. Bijelic, A.; Dobrov, A.; Roller, A.; Rompel, A. Binding of a Fatty Acid-Functionalized Anderson-Type Polyoxometalate to Human Serum Albumin. *Inorganic chemistry* **2020**, *59*, 5243-5246, doi:10.1021/acs.inorgchem.9b03407.
32. Maso, L.; Trande, M.; Liberi, S.; Moro, G.; Daems, E.; Linciano, S.; Sobott, F.; Covaceuszach, S.; Cassetta, A.; Fasolato, S.; et al. Unveiling the binding mode of perfluorooctanoic acid to human serum albumin. *Protein science : a publication of the Protein Society* **2021**, *30*, 830-841, doi:10.1002/pro.4036.
33. Yamasaki, K.; Kawai, A.; Sakurama, K.; Udo, N.; Yoshino, Y.; Saito, Y.; Tsukigawa, K.; Nishi, K.; Otagiri, M. Interaction of Benzbromarone with Subdomains IIIA and IB/IIA on Human Serum Albumin as the Primary and Secondary Binding Regions. *Molecular pharmaceutics* **2021**, *18*, 1061-1070, doi:10.1021/acs.molpharmaceut.0c01004.
34. Oltersdorf, T.; Elmore, S.W.; Shoemaker, A.R.; Armstrong, R.C.; Augeri, D.J.; Belli, B.A.; Bruncko, M.; Deckwerth, T.L.; Dinges, J.; Hajduk, P.J.; et al. An inhibitor of Bcl-2 family proteins induces regression of solid tumours. *Nature* **2005**, *435*, 677-681, doi:10.1038/nature03579.
35. Almogren, A.; Furtado, P.B.; Sun, Z.; Perkins, S.J.; Kerr, M.A. Purification, properties and extended solution structure of the complex formed between human immunoglobulin A1 and human serum albumin by scattering and ultracentrifugation. *Journal of molecular biology* **2006**, *356*, 413-431, doi:10.1016/j.jmb.2005.11.060.
